# Comparison of the shaping ability of GT^®^ Series X, Twisted Files and AlphaKite rotary nickel-titanium systems in simulated canals

**DOI:** 10.1186/1472-6831-13-72

**Published:** 2013-12-17

**Authors:** Raidan Ba-Hattab, Anne-Kathrin Pröhl, Hermann Lang, Dieter Pahncke

**Affiliations:** 1 Department of Operative Dentistry and Periodontology, Dental School University of Rostock, Strempelstr 13, 18057 Rostock, Germany

**Keywords:** AlphaKite, Canal shaping, GT^®^ Series X, Ni-Ti instruments, Simulated canals, Twisted files

## Abstract

**Background:**

Efforts to improve the performance of rotary NiTi instruments by enhancing the properties of NiTi alloy, or their manufacturing processes rather than changes in instrument geometries have been reported. The aim of this study was to compare in-vitro the shaping ability of three different rotary nickel-titanium instruments produced by different manufacturing methods.

**Methods:**

Thirty simulated root canals with a curvature of 35˚ in resin blocks were prepared with three different rotary NiTi systems: AK- AlphaKite (Gebr. Brasseler, Germany), GTX- GT^®^ Series X (Dentsply, Germany) and TF- Twisted Files (SybronEndo, USA).

The canals were prepared according to the manufacturers’ instructions. Pre- and post-instrumentation images were recorded and assessment of canal curvature modifications was carried out with an image analysis program (GSA, Germany).

The preparation time and incidence of procedural errors were recorded. Instruments were evaluated under a microscope with 15 × magnifications (Carl Zeiss OPMI Pro Ergo, Germany) for signs of deformation. The Data were statistically analyzed using SPSS (Wilcoxon and Mann–Whitney *U*-tests, at a confidence interval of 95%).

**Results:**

Less canal transportation was produced by TF apically, although the difference among the groups was not statistically significant. GTX removed the greatest amount of resin from the middle and coronal parts of the canal and the difference among the groups was statistically significant (*p* < 0.05). The shortest preparation time was registered with TF (444 s) and the longest with GTX (714 s), the difference among the groups was statistically significant (*p* < 0.05). During the preparation of the canals no instrument fractured. Eleven instruments of TF and one of AK were deformed.

**Conclusion:**

Under the conditions of this study, all rotary NiTi instruments maintained the working length and prepared a well-shaped root canal. The least canal transportation was produced by AK. GTX displayed the greatest cutting efficiency. TF prepared the canals faster than the other two systems.

## Background

It has been two decades since the first NiTi rotary files appeared on the market. Their introduction in endodontics has changed the way how root canal preparations are performed, enabling more complicated root canal systems to be shaped with fewer procedural errors [1]. The improvement in the instrument design with particular emphasis on tip configuration and cross sectional shape have reduced the prevalence and severity of canal aberrations [2].

Many rotary files have a body guided by a passive non-cutting tip that makes dentin cut more circumferentially. However, actively cutting files should never be extended beyond the root apex (accidently) to avoid the occurrence of apical zipping and perforation [1]. The presence of a positive blade rake angle enhances the cutting action of the instrument [3]. It also reduces the torsional load of the instruments. The flexibility of the instruments could be improved by reducing their residual core; consequently it is possible to increase the taper of the NiTi instruments [4]. Constant helical angles and a constant blade pitch which is the distance between two cutting edges can be adapted [5]. By varying these two parameters along the blade length, the cutting action and the ability to remove debris from the blades and prevent screwing can be improved [6].

In order to increase both the efficiency and safety of NiTi rotary files it has been suggested to improve the manufacturing process or using new alloys with superior mechanical properties such as (M-Wire alloy) [7]. This new NiTi alloy was developed in 2007 by Dentsply and is currently used for manufacturing GT series X and WaveOne instruments (Dentsply Tulsa-Dental Specialties). This alloy presents a higher fatigue resistance with a reduced risk for instrument fracture [8]. In 2008 SybronEndo (Orange, CA) developed new NiTi rotary files for root canal preparation called the Twisted Files (TF). These files have three new design methods of manufacturing, namely R-phase heat treatment, twisting of the metal, and special surface conditioning (deoxidation) [9]. It was reported to have a higher fracture resistance than traditional NiTi rotary files [7,10].

The recently introduced AlphaKite (Gebr. Brasseler, Germany), the new generation of Alpha system, is manufactured from conventional NiTi-alloy. The new system differs from the Alpha System in that all of the instruments have a kite-shaped cross-section, with one cutting angle and 3 supporting cutting angles. The instruments are physical vapor deposition-coated (PVD-coated) with a thin layer of TiN in order to increase their surface hardness. Previous studies have shown that the PVD technique significantly increases the cutting efficiency of NiTi instruments [11,12], enhances their wear resistance [13] and making smoother the superficial texture [14].

This study was conducted to compare in vitro the shaping ability of NiTi instruments produced by different manufacturing methods: M-wire [GT Series X (Dentsply, Germany)]; R-Phase [Twisted Files (SybronEndo, USA)] and Tin PVD-coated [AlphaKite instruments (Gebr. Brasseler, Germany)].

## Methods

Simulated curved canals made of clear polyester resin (Endo Training Block 02 taper, REFA 0177; Dentsply Maillefer, CH-1338 Ballaigues, Switzerland) with 35°. The diameter and the taper of all simulated canals were equivalent to an ISO standard size 15 root canal instrument. Canals were 17 mm long, the straight part being 12 mm and the curved part 5 mm. Prior to instrumentation, the specimens were divided into three experimental groups (n = 10) and were drilled on one side with a diamond bur to ensure repositioning accuracy in subsequent superimposition of the pictures and a coloring solution (Caries Marker, coloured caries indicator, VOCO, Cuxhaven, Germany) was injected into the canals.

The Blocks were placed with a black background in a reproducible position and the simulated canals were prepared with any of the three systems: AK, GTX and TF.

Pre- and post-instrumentation canal pictures were taken in a standardized manner using a digital camera EOS 400 Digital (Canon Inc., Tokyo, Japan) with a macro-objective “Tamron SP AF 60 mm F/2 Dill Macro 1:1” (Tamron Co., Ltd., Saitama, Japan) and stored directly in a computer.

The instruments were set into permanent rotation with a 6:1 reduction hand-piece (Sirona, Germany) powered by a torque-limited electric motor VDW Silver (VDW, Germany). The individual torque limit and rotational speed of each file which recommended by the manufacturers were entered and stored manually by the operator in the Dr’s Choice program.

FileCare (EDTA, VDW, München, Germany) was used as lubricant, and a total of 5 ml water was used repeatedly after the use of each instrument. Each instrument was used to enlarge one canal only. All of the canals were enlarged by the same operator who was experienced with all three systems. Once the instrument had achieved to the end of the canal and had rotated freely, it was removed.

The following instrumentation sequences were used with the different systems:

Group 1

TF instruments were used in a crown-down manner at a speed of 500 rpm as recommended by the manufacturer. A small assorted pack (25/.08, 25/.06, and 25/.04) was used. The preparation sequence was as follows: a 15, K-File was used to create a guide path; an 8% taper, size-25 instrument was used at (11 mm); a 6% taper, size-25 instrument was used at 14 mm; and a 4% taper, size-25 instrument was used at the full WL (17 mm).

Group 2

GTX instruments were used in a crown-down manner at a speed of 300 rpm as recommended by the manufacturer. The preparation sequence was as follows: a 15, K-File was used to create a guide path; a 6% taper, size-20 instrument was used at (11 mm); a 4% taper, size-20 instrument was used to the full WL (17 mm).

Group 3

AK instruments were used in a crown-down manner at a speed of 250 rpm as recommended by the manufacturer. The red assorted pack (25/.06, 25/.04, and 25/.02) was used. The preparation sequence was as follows: a 15, K-File was used to create a guide path; a 6% taper, size-25 instrument was used at (11 mm); a 4% taper, size-25 instrument was used at 14 mm; and a 2% taper, size-25 instrument was used at the full WL (17 mm).

### Assessment of canal preparation and analysis of data

Assessment of canal curvature modifications was carried out with the image analysis software (GSA Image Analyser Software development and Analytics Bansemer and Scheel GbR, Germany). A composite image of each canal was produced using the software from the pre- and final post-instrumented images. The area between canal configuration before and after instrumentation (material removed by instrumentation) was determined both for the inner and outer curvature using the Image Analyser program. Ten concentric circles spaced 1 mm apart were sectioned the composite image with their centers targeted over the apical end of the pre- instrumented canal, i.e. a radius of the first circle was 1 mm from the apical point of the canal and a radius of the last circle was 10 mm from the apical point. This resulted in a total of 20 segments (10 segments of the outer curvature and 10 segments of the inner curvature). The segments of all canals (material removed) were measured automatically with the GSA Image Analyser program in two dimensions as a surface area (mm^2^).

The cutting efficiency of instruments (the total amount of material removed at both the inner and outer canal walls) was evaluated in three parts of the root canal starting from apex: apical part which is the most curved part of the canal (segments 1–4), middle part (segments 5–7) and coronal part (segments 8–10).

Furthermore, based on the composite images, assessments were made according to the presence of different types of canal aberrations, such as apical zip, elbow, ledge and perforation. The canal aberrations were defined according to Thompson & Dummer [15].

After preparation of the blocks, all instruments were examined under a microscope with 15 × magnifications (Carl Zeiss OPMI Pro Ergo, Germany) for signs of deformation.

After preparation, canal length was measured using an ISO size-15 stainless steel hand K-file and Endo gauge. The K-file was placed in the canal and the length that it reached was marked by adjusting the rubber stop of the file to the upper surface of the resin block which served as reference surface. The change of working length was determined by subtracting the canal length after preparation from the original canal length (17 mm). The time for canal preparation including the total active instrumentation, instrument changes within the sequence, photography and irrigation was recorded.

Wilcoxon test was used to compare the material removed from the inner and outer canal walls of one group. To compare canal transportation among the groups, cutting efficiency and working time, Kruskal- Wallis and Mann–Whitney U-tests were used at a confidence interval of 95%) [SPSS, version 19.0 (IBM Corporation, USA)].

## Results

### Comparison of canal shape produced after instrumentation

The composite images enabled assessment of the material removed by preparation. Twenty segments were assessed along the canal length (10 segments of the outer curvature and 10 segments of the inner curvature). The results in Table 1 show that the removal of material over the length of the canal was not equal on the inner and outer curves. For all instruments significantly more material was removed on the outer wall than the inner wall in the apical and coronal parts of the canal except in segments 2 and 4 of TF and GTX groups respectively (*p* < 0.05). In the middle part of the canal more material was removed on the inner wall than the outer wall; the difference was statistically significant in segments 5 and 6 of GTX and TF groups and only in segment 6 of AK group (*p* < 0.05).

**Table 1:** Amount of material removed* (mm^**2**^**) for each instrument**

**Segments**	**1**	**2**	**3**	**4**	**5**	**6**	**7**	**8**	**9**	**10**
**GTX**										
**Outer wall**	0.06 ± 0.02	0.09 ± 0.02	0.11 ± 0.02	0.11 ± 0.02	0.08 ± 0.02	0.07 ± 0.02	0.16 ± 0.02	0.24 ± 0.01	0.26 ± 0.02	0.24 ± 0.02
**Inner wall**	0.02 ± 0.01	0.03 ± 0.02	0.04 ± 0.03	0.08 ± 0.04	0.15 ± 0.03	0.18 ± 0.02	0.17 ± 0.02	0.16 ± 0.02	0.15 ± 0.02	0.12 ± 0.03
***p- *** **value**	**0.011**	**0.008**	**0.005**	0.059	**0.005**	**0.005**	1.000	**0.005**	**0.005**	**0.005**
**TF**										
**Outer wall**	0.07 ± 0.04	0.06 ± 0.03	0.09 ± 0.03	0.12 ± 0.02	0.08 ± 0.01	0.07 ± 0.02	0.13 ± 0.02	0.19 ± 0.02	0.22 ± 0.02	0.22 ± 0.02
**Inner wall**	0.03 ± 0.02	0.04 ± 0.03	0.02 ± 0.03	0.04 ± 0.02	0.14 ± 0.02	0.18 ± 0.02	0.14 ± 0.02	0.11 ± 0.03	0.10 ± 0.03	0.09 ± 0.04
***p *-value**	**0.017**	0.083	**0.012**	**0.007**	**0.005**	**0.005**	0.836	**0.005**	**0.005**	**0.005**
**AK**										
**Outer wall**	0.07 ± 0.02	0.07 ± 0.02	0.10 ± 0.02	0.13 ± 0.02	0.10 ± 0.03	0.06 ± 0.01	0.09 ± 0.01	0.14 ± 0.01	0.15 ± 0.02	0.15 ± 0.02
**Inner wall**	0.04 ± 0.01	0.04 ± 0.01	0.04 ± 0.01	0.04 ± 0.02	0.11 ± 0.02	0.15 ± 0.02	0.10 ± 0.02	0.08 ± 0.01	0.08 ± 0.01	0.08 ± 0.02
***p- *** **value**	**0.007**	**0.01**	**0.005**	**0.005**	0.310	**0.005**	**0.281**	**0.005**	**0.005**	**0.005**

*Means ± standard deviations.

Values in bold are statistically significant.

Table 2 presents the result comparing the three groups and demonstrates that in segments (1–6) no statistically significant difference among the groups was found in removing material from the outer canal wall. In the GTX group, significantly (*p* < 0.05) more outer canal wall was removed in segments (7–10) than in the TF and AK groups. In the inner canal wall, there was no statistically significant difference among the groups in removing material in segments (1–3). GTX significantly (*p* < 0.05) removed more material than the other two systems in segments 4, 7, 8 and 9. In segments 5, 6 and 10, the difference between GTX and TF was statistically not significant.

**Table 2:** Comparison among the instruments of the amount of material removed* (mm^**2**^**) from canal walls**

**Segments**	**1**	**2**	**3**	**4**	**5**	**6**	**7**	**8**	**9**	**10**
**Outer wall**										
**GTX**	0.06 ± 0.02^a^	0.09 ± 0.02^a^	0.11 ± 0.02^a^	0.11 ± 0.02^a^	0.08 ± 0.02^a^	0.07 ± 0.02^a^	0.16 ± 0.02^a^	0.24 ± 0.01^a^	0.26 ± 0.02^a^	0.24 ± 0.02^a^
**TF**	0.07 ± 0.04^a^	0.06 ± 0.03^a^	0.09 ± 0.03^a^	0.12 ± 0.02^a^	0.08 ± 0.01^a^	0.07 ± 0.02^a^	0.13 ± 0.02^b^	0.19 ± 0.02^b^	0.22 ± 0.02^b^	0.22 ± 0.02^b^
**AK**	0.07 ± 0.02^a^	0.07 ± 0.02^a^	0.10 ± 0.02^a^	0.13 ± 0.02^a^	0.10 ± 0.03^a^	0.06 ± 0.01^a^	0.09 ± 0.01^c^	0.14 ± 0.01^c^	0.15 ± 0.02^c^	0.15 ± 0.02^c^
**Inner wall**										
**GTX**	0.02 ± 0.01^a^	0.03 ± 0.02^a^	0.04 ± 0.03^a^	0.08 ± 0.04^a^	0.15 ± 0.03^a^	0.18 ± 0.02^a^	0.17 ± 0.02^a^	0.16 ± 0.02^a^	0.15 ± 0.02^a^	0.12 ± 0.03^a^
**TF**	0.03 ± 0.02^a^	0.04 ± 0.03^a^	0.02 ± 0.03^a^	0.04 ± 0.02^b^	0.14 ± 0.02^a^	0.18 ± 0.02^a^	0.14 ± 0.02^b^	0.11 ± 0.03^b^	0.10 ± 0.03^b^	0.09 ± 0.04^a.b^
**AK**	0.04 ± 0.01^a^	0.04 ± 0.01^a^	0.04 ± 0.01^a^	0.04 ± 0.02^b^	0.11 ± 0.02^b^	0.15 ± 0.02^b^	0.10 ± 0.02^c^	0.08 ± 0.01^c^	0.08 ± 0.01^b^	0.08 ± 0.02^b^

*means ± standard deviations.

^a,b,c^ There are no significant differences between the groups with the same letters.

### Total amount of material removed

The cutting efficiency of the instruments, which was represented by the total amount of material removed at both the inner and outer canal walls (20 segments of root canal), is detailed in Table 3, which shows that GTX instruments significantly removed more resin from the middle and coronal parts of the canal (*p* < 0.000). The difference among the instruments in apical part of the canal was statistically not significant (*p* ≥ 0.05).

**Table 3:** Material removed* (mm^**2**^**) in three parts of root canals**

**Instruments**	**Apical part**	**Middle part**	**Coronal part**	**Total amount of material removed**
**GT® series X**	0.07 ± 0.04^a^	0.13 ± 0.05^a^	0.19 ± 0.06^a^	0.39 ± 0.06^a^
**Twisted files**	0.06 ± 0.04^a^	0.12 ± 0.04^b^	0.15 ± 0.06^b^	0.33 ± 0.06^a,b^
**AlphaKite**	0.07 ± 0.04^a^	0.11 ± 0.06^c^	0.11 ± 0.04^c^	0.29 ± 0.03^b^
***p- *** **value**	**0.274**	**0.000**	**0.000**	**0.046**

* Values of means of the total amount of material removed ± Standard deviation.

^a,b,c^ There are no significant differences between the groups with the same letters.

### Canal aberration and loss of working length

No loss of working length or canal aberration was recorded in any of the groups. All canals remained patent after instrumentation (i.e. none of the canals became blocked with resin chips).

### Working time

The shortest mean preparation time was recorded when TF instruments were used (444 seconds) followed by AK (528 seconds) and GTX (714 seconds) consequently. The difference among the three systems was statistically significant (*p* < 0.05).

### Working safety

During the preparation of the canals no instrument fractured. Eleven instruments of TF system (nine of size 25/.08 and two of size 25/.06 taper) and only one instrument of AK (size-25/.04) were deformed.

## Discussion

The purpose of this study was to compare the shaping ability of three different rotary NiTi instruments produced by different manufacturing methods in simulated root canals. The use of resin blocks provides an appropriate evaluation of the preparation outcome and instrument performance [16]. The changes in the canal shape with resin blocks are recognized faster than dentin due to its transparency. However, the action of the instrument in a real root canal differs from that of simulated canals in resin blocks due differences in surface texture, hardness and cross-section [17].

Canal transportation demonstrates the straightening tendency of the file as it prepares the canal. The NiTi files that we have used in our study are pseudo-elastic. This means that the files prepare curved canals and do straighten within the canal as they prepare [18]. In our study, the three tested rotary systems resulted in canal transportation at most examined levels, a finding which is consistent with other studies that show that canal transportation occurs mostly in curved canals at the outer wall of the apical portion of the canal and the inner aspect of the mid-root of the canal [19,20].

In this study, TF instruments produced the least apical transportation although the difference among the three groups was statistically not significant. This finding is in agreement with what has been reported by Gergi et al. [21]. They concluded that TF instruments caused less apical transportation than PathFile-ProTaper instruments in extracted teeth. Fayyad and Elgendy [22] found that TF system was to cut dentin efficiently with more uniform cutting than ProTaper system in extracted teeth. Furthermore El Batouty and Elmallah [23] suggested that TF instruments showed a greater tendency to preserve the curvature of curved canals in extracted teeth than K3 instruments. According to these authors, the enhanced capacity of TF instruments to shape the canal might be attributed to the new manufacturing method of R- Phase technology which makes them more flexible than other NiTi instruments that manufactured by grinding process.

Concerning GTX instruments, it is interesting to note that these instruments removed nearly the same amount of material apically as did AK and TF instruments although they have smaller ISO size-20/.04. In the middle and coronal parts of the canal, they showed a cutting efficiency superior to those of TF and AK instruments.

GTX system is a modified version of the ProSystem GT and characterized by M-wire NiTi technology. The instruments have more open blade angles, variable-width lands, and a 1-mm maximum shank diameter since the variable-width lands produce large chips space between the cutting flutes. Therefore, it is supposed that the enhancement of the cutting efficiency of GTX is attributed to the design of their radial lands [24].

Tabatabaei [25] found that ProSystem GT produce more canal displacement than GTX in extracted teeth. Hashem et al. [26] observed that GTX removed more dentin than TF and Revo-S but with no statistical significance. They also concluded that TF system remained more centered and producing less canal transportation than RS, GTX and ProTaper.

AK instruments removed material significantly less than those of TF and GTX instruments in the middle and coronal parts of the canal. AK instruments possess only one cutting angle and 3 supporting cutting angles, with a kite-type cross-sectional design. This large cross sectional design might result in chips space smaller than other instruments and consequently less resin removal capability. Al-dameh [27] suggested that AlphaKite and BioRaCe instruments produced moderately well centered preparations in extracted teeth with minimal transportation and were relatively safe.

TF instruments prepared the canals significantly faster than the other two systems. The operator factors and the preparation techniques influence the working time more than the instruments themselves [28]. Therefore, caution must be taken when comparing the findings of different studies as the individual variations cannot be exactly estimated [29].

Detection of early signs of metal fatigue in nickel-titanium instruments are not usual; whereas deformation of stainless steel files serves as a warning of upcoming fracture [30]. In the present study, visible inspection of all instruments showed deformation of eleven instruments of TF, further examination of the instruments under microscope showed deformation of only one AK instrument. Therefore, although visible inspection is to be advisable, it would not seem to be the optimal way for the evaluation of nickel-titanium instruments in order to avoid fracture. However the greater resistance of TF instruments to cyclic fatigue over the traditional NiTi instruments produced by grinding had been proved in various studies [31-34].

## Conclusion

Within the limitations of this in vitro study, all rotary nickel titanium instruments maintained the working length and prepared a well-shaped root canal without aberration. AlphaKite system produced the least canal transportation. GT Series X system displayed the greatest amount of material removal. Twisted Files system prepared the canals faster than the other two systems.

## Competing interests

The authors declare that they have no competing interests.

## Authors’ contributions

RBH performed the experiment work and involved in the analysis, interpretation of data, report writing and manuscript writing. DP involved in the design of the experiment, interpretation of data and manuscript review. AKP and HL contributed to data interpretation and preparation of the manuscript. All authors have read and approved the final version of the manuscript.

## Pre-publication history

The pre-publication history for this paper can be accessed here:

http://www.biomedcentral.com/1472-6831/13/72/prepub
